# Multivariable prognostic modelling to improve prediction of colorectal cancer recurrence: the PROSPeCT trial

**DOI:** 10.1007/s00330-024-10803-7

**Published:** 2024-06-05

**Authors:** Vicky Goh, Susan Mallett, Victor Boulter, Robert Glynne-Jones, Saif Khan, Sarah Lessels, Dominic Patel, Davide Prezzi, Manuel Rodriguez-Justo, Stuart A. Taylor, Richard Beable, Margaret Betts, David J. Breen, Ingrid Britton, John Brush, Peter Correa, Nicholas Dodds, Joanna Dunlop, Sofia Gourtsoyianni, Nyree Griffin, Antony Higginson, Andrew Lowe, Andrew Slater, Madeline Strugnell, Damian Tolan, Ian Zealley, Steve Halligan, For PROSPECT investigators, Vicky Goh, Victor Boulter, Robert Glynne-Jones, Saif Khan, Sarah Lessels, Dominic Patel, Davide Prezzi, Manuel Rodriguez-Justo, Richard Beable, Margaret Betts, Ingrid Britton, Joanna Dunlop, Sofia Gourtsoyianni, Antony Higginson, Andrew Slater, Steve Halligan, David J. Breen, John Brush, Peter Correa, Nicholas Dodds, Nyree Griffin, Andrew Lowe, Susan Mallett, Michelle McDermaid, Amjad Mohammad, Colin Oliver, Madeline Strugnell, Stuart A. Taylor, Damian Tolan, Biju Thomas, Ian Zealley

**Affiliations:** 1 School of Biomedical Engineering & Imaging Sciences, King’s College London, London, UK; 2 Department of Radiology, Guys and St. Thomas’ NHS Foundation Trust, London, UK; 3 Centre for Medical Imaging, Division of Medicine, University College London, London, UK; 4 Patient Representative, Mount Vernon Cancer Centre, Northwood, UK; 5 Mount Vernon Cancer Centre, Northwood, UK; 6 Research Department of Pathology, UCL Cancer Institute, University College London, London, UK; 7 Scottish Clinical Trials Research Unit, Public Health Scotland, Edinburgh, UK; 8 Department of Radiology, Portsmouth Hospitals University NHS Trust, Portsmouth, UK; 9 Department of Radiology, Oxford University Hospitals NHS Foundation Trust, Oxford, UK; 10 Department of Radiology, University Hospital Southampton NHS Foundation Trust, Southampton, UK; 11 Department of Radiology, University Hospitals North Midlands NHS Trust, Stoke-On-Trent, UK; 12 Department of Radiology, Western General Hospital, NHS Lothian, Edinburgh, UK; 13 Department of Oncology, University Hospitals Coventry and Warwickshire NHS Trust, Coventry, UK; 14 Department of Radiology, Jersey General Hospital, St. Helier, Jersey; 15 Department of Radiology, Musgrove Park Hospital, Somerset NHS Foundation Trust, Taunton, UK; 16 Department of Radiology, Royal Cornwall Hospitals NHS Trust, Truro, UK; 17 Department of Radiology, St James’s University Hospital, Leeds Teaching Hospitals NHS Trust, Leeds, UK; 18 Department of Radiology, Ninewells Hospital, NHS Tayside, Dundee, UK; 19 Department of Radiology, Bradford Teaching Hospitals NHS Foundation Trust, Bradford, UK; 20 Department of Radiology, University Hospitals Coventry and Warwickshire NHS Trust, Coventry, UK

**Keywords:** Prognostic model, Neoplasms/primary, Large bowel, Angiogenesis, CT-perfusion

## Abstract

**Objective:**

Improving prognostication to direct personalised therapy remains an unmet need. This study prospectively investigated promising CT, genetic, and immunohistochemical markers to improve the prediction of colorectal cancer recurrence.

**Material and methods:**

This multicentre trial (ISRCTN 95037515) recruited patients with primary colorectal cancer undergoing CT staging from 13 hospitals. Follow-up identified cancer recurrence and death. A baseline model for cancer recurrence at 3 years was developed from pre-specified clinicopathological variables (age, sex, tumour-node stage, tumour size, location, extramural venous invasion, and treatment). Then, CT perfusion (blood flow, blood volume, transit time and permeability), genetic (RAS, RAF, and DNA mismatch repair), and immunohistochemical markers of angiogenesis and hypoxia (CD105, vascular endothelial growth factor, glucose transporter protein, and hypoxia-inducible factor) were added to assess whether prediction improved over tumour-node staging alone as the main outcome measure.

**Results:**

Three hundred twenty-six of 448 participants formed the final cohort (226 male; mean 66 ± 10 years. 227 (70%) had ≥ T3 stage cancers; 151 (46%) were node-positive; 81 (25%) developed subsequent recurrence. The sensitivity and specificity of staging alone for recurrence were 0.56 [95% CI: 0.44, 0.67] and 0.58 [0.51, 0.64], respectively. The baseline clinicopathologic model improved specificity (0.74 [0.68, 0.79], with equivalent sensitivity of 0.57 [0.45, 0.68] for high vs medium/low-risk participants. The addition of prespecified CT perfusion, genetic, and immunohistochemical markers did not improve prediction over and above the clinicopathologic model (sensitivity, 0.58–0.68; specificity, 0.75–0.76).

**Conclusion:**

A multivariable clinicopathological model outperformed staging in identifying patients at high risk of recurrence. Promising CT, genetic, and immunohistochemical markers investigated did not further improve prognostication in rigorous prospective evaluation.

**Clinical relevance statement:**

A prognostic model based on clinicopathological variables including age, sex, tumour-node stage, size, location, and extramural venous invasion better identifies colorectal cancer patients at high risk of recurrence for neoadjuvant/adjuvant therapy than stage alone.

**Key Points:**

- *Identification of colorectal cancer patients at high risk of recurrence is an unmet need for treatment personalisation*.
- *This model for recurrence, incorporating many patient variables, had higher specificity than staging alone*.
- *Continued optimisation of risk stratification schema will help individualise treatment plans and follow-up schedules*.

## Introduction

Up to 50% of patients with colorectal cancer ultimately die from metastatic disease, occult at diagnosis [1]. Adjuvant chemotherapy following surgery aims to eradicate micrometastases but offering this indiscriminately may be overtreatment. Streamlining patients who should receive adjuvant therapy turns on prognosis, based largely on pathological tumour and nodal stage [2, 3]. However, patients with identical stage tumours can experience widely divergent survival outcomes: 5-year survival varies between 63–87% for American Joint Committee on Cancer (AJCC, tumour-node-metastasis (TNM) stage grouping) stage II; and stage IIIA survival may exceed stage IIB/IIC [4, 5]. Also, the shift towards neoadjuvant therapy for colon as well as rectal cancer has highlighted a need for better preoperative identification of high-risk patients [6, 7].

Multivariable prognostic models combine multiple factors to estimate the risk of future outcome(s). While models predicting colorectal cancer outcomes are available in different clinical settings [8, 9], they are not used widely. A criticism has been that they do not include promising predictors despite recent research around imaging, genetic, and immunohistochemical biomarkers. It was hypothesised that a baseline multivariable model to predict the recurrence of colorectal cancer could be improved by the addition of more novel, promising imaging, genetic, and pathological markers of angiogenesis and hypoxia. To achieve this, a prospective multicentre trial was designed specifically to develop a prognostic model of disease-free survival. The aim was to investigate promising CT perfusion imaging and genetic and immunohistochemical markers to improve the prediction of colorectal cancer recurrence.

## Methods

### Study design and participants

PROSPeCT (Improving PRediction Of metaStatic disease in Primary coloreCTal cancer) was a prospective, multicentre, cohort trial (ISRCTN: 95037515; REC: 10/H0713/84), conducted according to the principles of good clinical practice, and run by a clinical trials unit. Independent oversight was provided by the Data Monitoring and Trial Steering Committees. Research is reported according to transparent reporting of a multivariable prediction model for individual prognosis or diagnosis guidelines [10].

Consecutive adult participants were recruited from 13 university and community hospitals between November 2011 and 2016. Eligible patients had histologically proven or suspected (endoscopy and/or imaging) primary colorectal cancer. Participants were identified via outpatient clinics, imaging requests, endoscopy lists, and tumour board meetings. Exclusions were polyp cancers; metastases at staging; contraindication to intravenous contrast agent; an invisible tumour on CT; pregnancy; concurrent cancer, and a final non-cancer diagnosis. All participants provided written informed consent.

### CT imaging procedures

Participating centres underwent training and quality control for data acquisition [11]. In addition to a staging CT, participants underwent CT perfusion of the primary tumour. This was performed on the same occasion. The CT perfusion dynamic acquisition commenced 5 s following intravenous contrast injection (> 300 mg/mL iodine; 50 mLs at 5 mL/s followed by a saline chaser), with images at 1.5-s intervals for 45 s, then at 15-s intervals for 75 s. This was followed by the contrast-enhanced staging CT which was performed according to the institutional standard protocol (Supplementary Table 1, CT acquisition parameters).

CT perfusion scans were analysed by 25 designated local radiologists (with ≥ 5 years of subspecialty experience), after central training for software familiarisation and analysis. All had a subspecialty interest in gastrointestinal imaging. Radiologists used commercially available software provided by their CT vendor. Kinetic models included the distributed parameter model; Patlak analysis; deconvolution; and maximum slope.

Using the corresponding software, radiologists defined the arterial input function; defined when the contrast the first pass had ended; and outlined the tumour contour. This was achieved by placing a fixed-size (10 mm^2^) circular region-of-interest (ROI) in the largest visualised artery; marking the time-point on the displayed attenuation-time curve when the lower inflection point of the curve was reached; and outlining the tumour contour using a free-hand ROI, encompassing the largest tumour area possible but taking care to avoid non-tumoural tissue (area ranging from 9.5 mm^2^ to 2981 mm^2^).

This generated the following vascular parameters: regional blood flow, blood volume, mean transit time, or permeability surface area product (dependent on vendor software). Perfusion variables were recorded on a case report form that also detailed tumour dimensions and location. CT TNM stage was also determined. Following imaging data transfer, CT analysis was repeated centrally by three radiologists with 5–18 years of experience in CT perfusion, using the same software used locally, unaware of prior measurements and outcomes.

### Pathology procedures

For patients undergoing surgery, pathological staging was performed by pathologists at the participating institutions. Tumour staging was based on the fifth edition of the AJCC TNM staging classification as defined in the trial protocol and recorded on a case report form. Formalin-fixed paraffin-embedded blocks were also transferred centrally for additional analysis by two subspecialty pathologists who assessed: DNA mismatch repair (MMR) protein status (via expression of MLH1, MSH2, MSH6, and PMS2); CD105 microvessel density; vascular endothelial growth factor (VEGF) expression; glucose transporter protein (GLUT-1) expression; hypoxia-inducible factor-α (HIF-1α) expression. Tissue sections were batch-stained (Bond-Rx^m^, Leica Biosystems; Bond Polymer Refine Detection), scanned at ×20 magnification (Hamamatsu Nanozoomer 2.0 RS), and displayed on an LCD monitor with standardised contrast, focus, saturation, and white balance standardisation. VEGF, Glut-1, and HIF-1α were scored on staining intensity and proportion of positive cells according to previously published systems: VEGF and Glut-1 expression was calculated by combining staining intensity (0–3) with the percentage of positive cells (0–4), and HIF-1α expression on combined cytoplasmic and nuclear staining (range, 0–6). Visiopharm software evaluated CD105 staining. DNA was extracted for somatic mutation analysis (KRAS, BRAF, PIK3CA, pTEN, APC, and HRAS), and quality and quantification were assessed (Agilent Tapestation 2200). Preparation and sequencing used Life Technologies Ion Torrent, and analysed using Integrative Genomics Viewer.

### Clinical management decisions and follow-up

Standard clinical, radiological, and pathological investigations were interpreted and discussed at the tumour board meeting at each participating institution and treatment decisions were undertaken as per usual clinical practice. For the primary outcome, participants were followed for 36 months (or death if sooner) and findings from outpatient visits, surveillance and/or symptomatic CT, carcinoembryonic antigen, and any other relevant investigations were recorded.

### Data collation and outcomes

The clinical trials unit collated and entered data into a bespoke database, and missing fields or possible inaccuracies were queried. Baseline data included participant demographics, date and results of staging investigations, and stage and planned management determined at the tumour board meeting. The date of any recurrence or death was recorded. Recurrence was considered alongside histology from any further resections or biopsies. Assessment of recurrence was blinded to genetic and immunochemistry results, and to principal component weighting (PCA) for CT perfusion variables.

### Statistical analysis

The primary outcome was to improve the prediction of recurrence or death by developing a model of disease-free survival, superior to current practice. A recurrence event was defined as metastasis, local recurrence/new primary, and/or any death (recorded as the primary event in patients with other simultaneous events). Outcomes were based on Nelson-Aalen cumulative hazard estimates of pre-specified risk groups at 3 years, using time-to-event models. Predictions by risk group were compared via (i) differences in sensitivity and specificity and (ii) a hypothetical population of 1000 participants diagnosed with colorectal cancer, to compare different models.

Modelling strategy: a best “baseline” model (Model A) was developed from prespecified standard clinical and pathological variables, namely TN stage, age, sex, tumour location and size, EMVI, and planned treatment. Univariable significance was not used to select variables. In order to determine the benefit (if any) of promising biomarkers, these were added to the standard model to create new models as follows: Model B (local CT perfusion variables via composite principal components analysis (PCA) score); Model D (simplest single local CT perfusion variable); Model E (central CT perfusion variables via PCA score); and Model F (pathology variables: immunohistochemical angiogenesis and hypoxia markers plus somatic mutations).

Prediction of all models was compared to standard TN staging (rule C; “clinical rule”), with “high risk” patients defined by stage III AJCC stage grouping and “low risk” patients defined by stage I/II [12]. In order to mirror model usage in clinical practice, imaging staging was used in the standard model for patients receiving neoadjuvant therapy or in whom surgery was not planned; imaging staging is deemed accurate when compared with pathological staging [13]. The pathological stage was used in patients having surgery first.

Model methods: The standard model was a Wiebull parametric (STATA “stpm2”). Risk groups were pre-specified based on tertile groups for each model; i.e. high risk = top tertile; medium risk = mid tertile; low risk = bottom tertile. Model performance was presented using Kaplan–Meier plots of risk groups (high vs medium/low risk, and high/medium vs low), with 95% confidence intervals (CI) and risk tables. Standard measures of discrimination and calibration were also calculated, including c-index and calibration slope. Internal validation using bootstrapping (100 repeats) was used to assess over-optimism. Additional details regarding sample size, powering, and prognostic modelling are presented in the Supplementary material.

## Results

### Participants

**Fig. 1:**
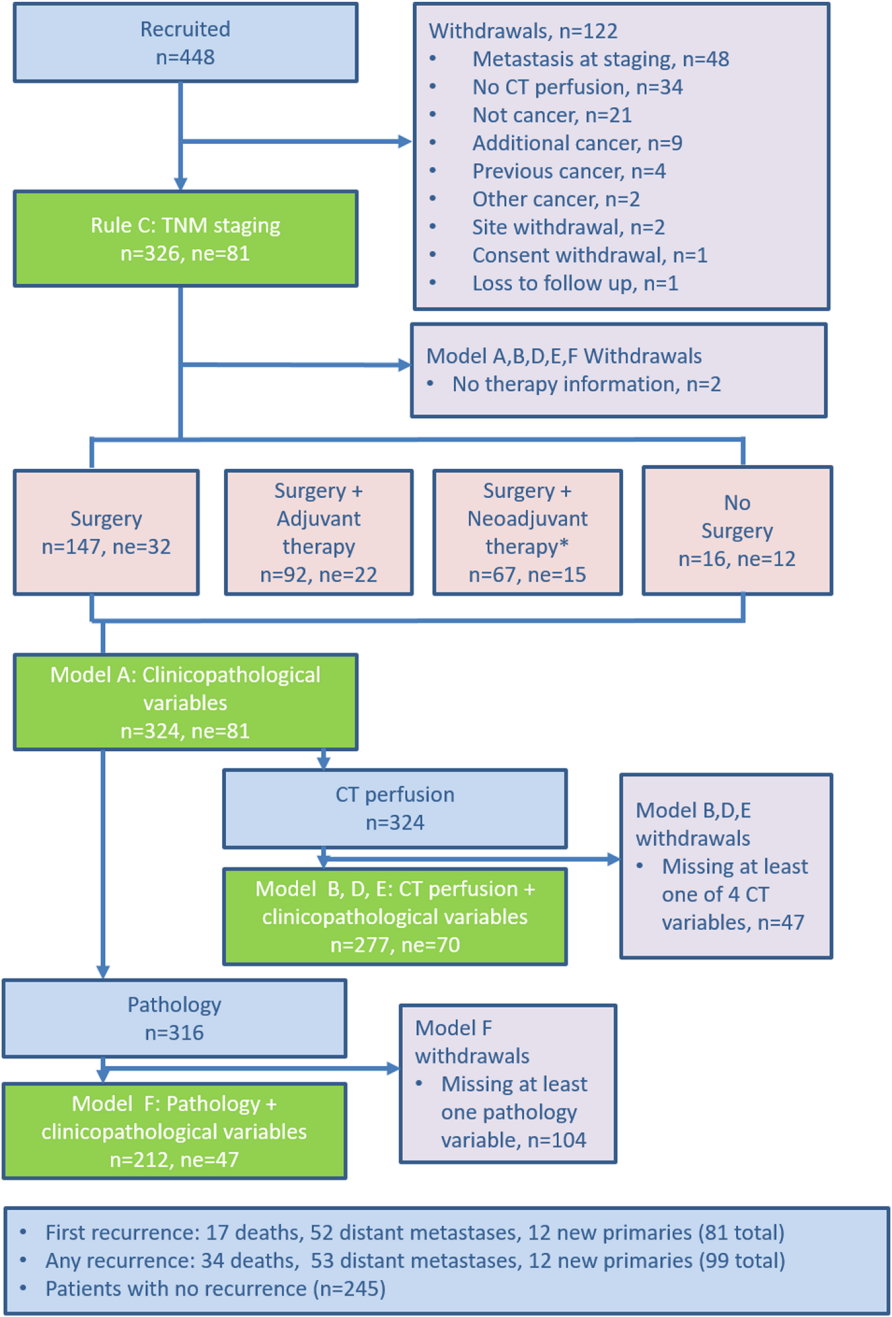
Flowchart showing participant flow through the trial. n, number; ne, not evaluable. *Note: neoadjuvant therapy included chemoradiotherapy, radiotherapy alone and chemotherapy alone

The participant flowchart is shown in Fig. 1. Baseline participant and tumour characteristics are shown in Table 1. Of 448 participants who were recruited, 122 (27%) were withdrawn, leaving 326 participants in the final cohort (226 male, 100 female; mean ± SD age 66 ± 10.7 years. 143/326 (44%) had colon and 183/326 (56%) rectal cancer (including rectal cancers extending into the rectosigmoid region). Surgery was performed ultimately in 308/326 (94%), of whom 92/308 (30%) had adjuvant therapy, and 67/308 (22%) had neoadjuvant therapy. Following neoadjuvant treatment, there were 12/183 (7%) rectal cancer complete responders; 5/12 received no further treatment. There was no therapy information for two participants.

**Table 1:** Baseline characteristics of final participant cohort (*n* = 326)

Variable		Recurrence	No recurrence	Total
Gender	Female	25 (31)	75 (31)	100 (31)
	Male	56 (69)	170 (69)	226 (69)
Age (years)	Mean ± SD	70 ± 10	65 ± 11	66 ± 11
Tumour location	Right colon	24 (27)	55 (23)	79 (24)
	Left colon	22 (24)	52 (21)	74 (20)
	Rectum	45 (49)	138 (56)	183 (56)
Tumour size (mm)	Median [IQR] Min, max	40 [30, 50] 18, 75	40 [30, 50] 10, 150	40 [30, 50] 10,150
T stage	T1	1 (1)	11 (5)	12 (4)
	T2	12 (15)	75 (31)	87 (27)
	T3	45 (56)	138 (55)	183 (55)
	T4	23 (28)	21 (9)	44 (14)
N stage	N0	38 (47)	137 (56)	175 (54)
	N1	25 (32)	73 (30)	98 (30)
	N2	18 (21)	35 (14)	53 (16)
Venous invasion	Present	36 (44)	57 (23)	93 (28)
	Absent	45 (56)	188 (77)	233 (72)
Resection margin	Involved	3 (4)	12 (5)	15 (4)
	Clear	54 (67)	183 (75)	237 (73)
	Missing data	24 (29)	50 (20)	74 (23)

Data provided are number (%) unless stated otherwise. Percentages provided are according to column data rather than row data. This allows comparison of the variable proportions within the recurrence, or no recurrence or total groups

Imaging staging was used in 83 (26%) and pathological staging in 241 cases (74%) for modelling. Most cancers were locally advanced (227/326 ≥ T3, 70%); 151/326 (46%) were node-positive (≥ N1 stage, Table 1). 93/326 (29%) had a venous invasion. The resection margin was positive in 15 (6%) of 252 with recorded data. Ultimately, there were 81 events over 3 years: 31 (39%) in year 1; 29 (36%) in year 2; and 21 (25%) in year 3. Fifty-two (64%) developed metastasis. Twelve (14%) developed new primaries. Seventeen (22%) died. There was venous invasion in a higher proportion of participants with recurrence (36/81, 44%) than without (57/245, 23%), with a significant relationship in both univariable and multivariable analysis with standard clinical variables (Supplementary material).

### CT perfusion analysis

CT perfusion measurements from participating sites showed no apparent difference for participants with and without recurrence at local and central review (Supplementary Table 2).

### Immunohistochemical and somatic mutation analysis

Immunohistochemical and somatic mutation analysis split by participants with and without recurrence are shown in Supplementary Tables 3 and  4. Distributions of HIF-1 α, VEGF, and GLUT-1 scores were similar across both groups (Supplementary Table 3). Participants with KRAS wild type had the largest difference in the proportion of participants with recurrence (34/62, 55%) than without (96/208, 46%) (Supplementary Table 4). Univariable and multivariable hazard ratios showed that genetic and immunohistochemistry variables were not associated with recurrence for all variables included in modelling (Supplementary Tables 5–8).

### Prognostic modelling

**Fig. 2:**
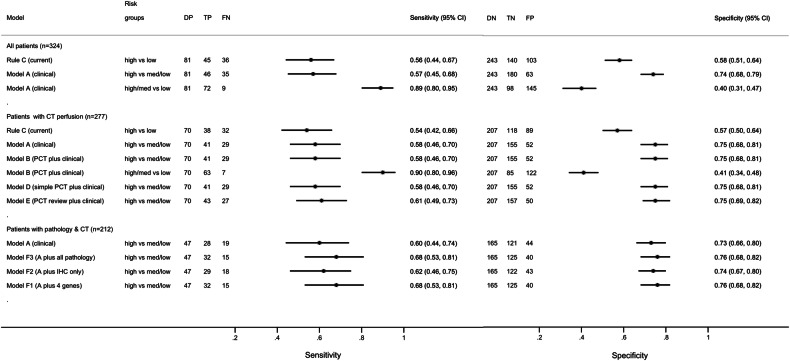
Forest plot of sensitivity and specificity, and 95% CI, for disease recurrence for standard AJCC tumour-node staging (rule C) compared to the baseline clinicopathological model (model A). Data are also shown for the various models incorporating CT perfusion imaging markers, or genetic/immunohistochemical markers to the baseline clinicopathological model. AJCC, American Joint Committee on Cancer; PCT, CT perfusion; IHC, immunohistochemistry

Sensitivity and specificity for standard AJCC TNM staging for predicting recurrence were 0.56 (95% CI: 0.44, 0.67) and 0.58 (95% CI: 0.51,0.64), respectively (Fig. 2). The equation for the best model of clinicopathological variables (TN stage, sex, age, tumour location and size, EMVI, and treatment; Model A) is presented in Supplementary material. This model was used at two operating points: at “high” vs “medium/low” risk, specificity improved over staging alone to 0.74 (95% CI: 0.68, 0.79) but with equivalent sensitivity of 0.57 (95% CI: 0.45, 0.68). At “high/medium” vs “low” risk, sensitivity over staging improved to 0.89 (95% CI: 0.80, 0.95) but with diminished specificity of 0.40 (95% CI: 0.31, 0.47).

The addition of CT perfusion to the baseline clinicopathological model (Model A) did not improve prediction substantially over and above this model alone (Fig. 2, Model B–E). For example, for Model B (i.e. Model A + local CT perfusion variables) sensitivity and specificity at the “high” vs “medium/low” risk threshold was 0.58 (95% CI: 0.46, 0.70) and specificity 0.75 (95% CI: 0.68, 0.81).

The addition of genetic and immunohistochemical markers to the baseline clinicopathological model (Model A) also did not improve prediction substantially over and above the standard model (Fig. 2, Models F1–F3). For example, for Model F3 (i.e. Model A + all pathology variables), sensitivity and specificity at the “high” vs “medium/low” risk threshold was 0.68 (95% CI: 0.53, 0.81) and specificity 0.76 (95% CI: 0.68, 0.82).

**Fig. 3:**
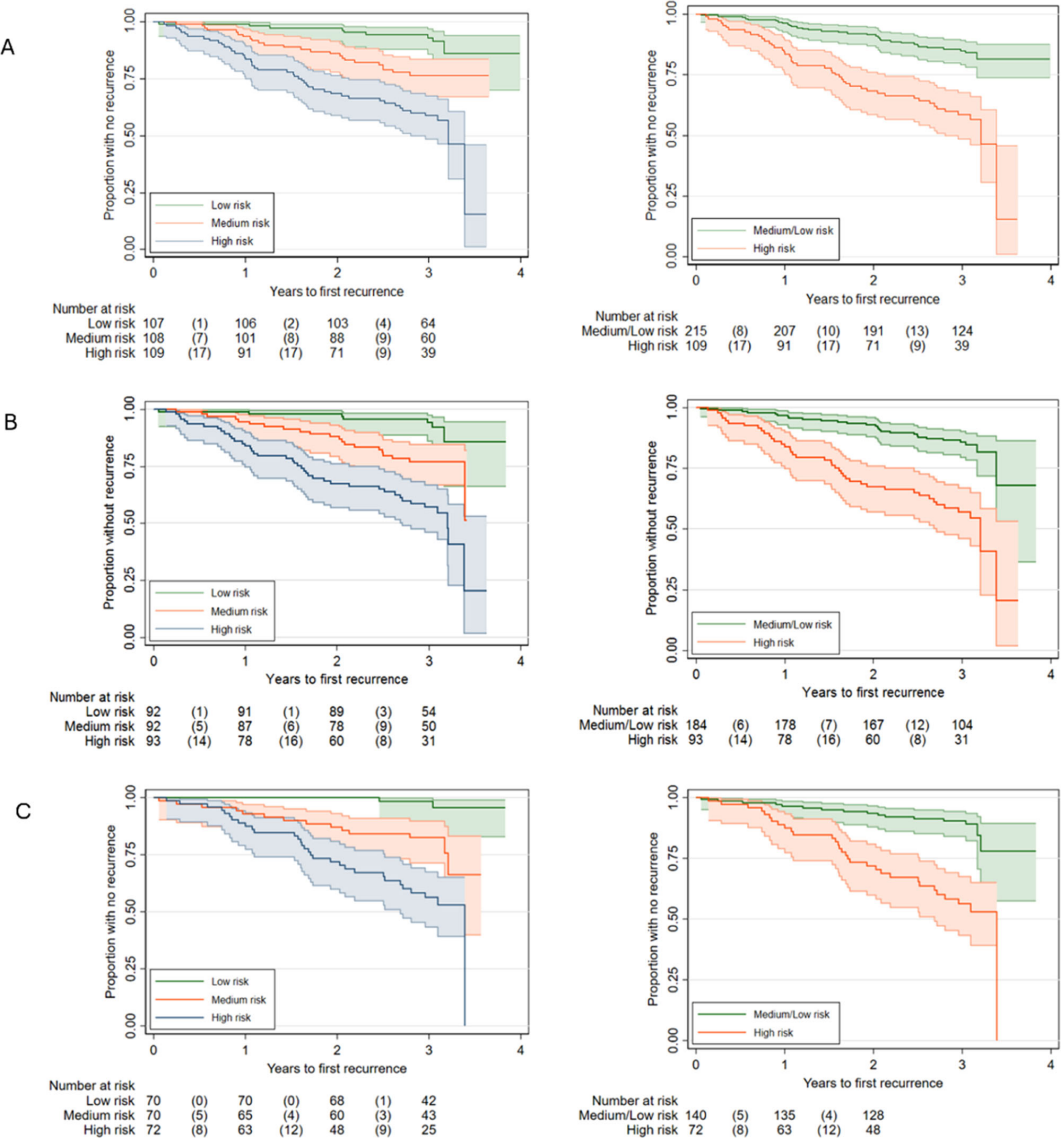
Kaplan–Meier (K–M) curves for Model A (baseline clinicopathological variables), Model B (baseline + CT perfusion variables), and Model F (baseline + pathology variables). **A** K–M curve for standard clinicopathological variables (Model A) at three different risk groupings defined by the prediction index. The graphs shown are respectively: high vs medium vs low risk and high vs medium/low risk. The high-risk group consisted the 33% of participants with the highest prediction. **B** K–M curves for Model B (i.e. Model A plus CT perfusion variables assessed at local sites). The distribution of risk groupings is similar to Model A alone, indicating that model prediction is not improved significantly by the addition of CT perfusion variables derived by local site analysis. **C** K–M curves for Model F (i.e. Model A + all novel immunohistochemical and genetic marker variables). The distribution of risk groupings is similar to Model A alone, indicating that model prediction is not improved significantly by the addition of novel pathology variables. Note: primary outcome: data beyond the 3-year time-point is sparse and should not be over-interpreted

Kaplan–Meier curves for the predictive performance of the baseline model A and other selected models are shown in Fig. 3. Kaplan–Meier curves for the predictive performance of standard staging are shown in Supplementary Fig. 1. Table 2 summarises prediction measures of discrimination and calibration for all models. Ultimately, the addition of the previously published promising markers failed to improve the prediction of the clinicopathological model meaningfully.

**Table 2:** Summary of model performance for the different models. Model A consisting of standard clinicopathological variables demonstrated the best performance with the lowest values for AIC and BIC. The addition of CT (Model B, D, D2, and E) and pathology (Model F1–F3) variables to Model A did not improve baseline performance

Model	Log-likelihood	*c* statistic	*R*^2^	Adj *R*^2^	*D* statistic (SE)	Adj *D* statistic (SE)	Calibration slope	Model degrees freedom	AIC	BIC
A: baseline clinical variables	128.4	0.76 (0.70, 0.82)	0.38	0.37	1.61 (0.26)	1.57 (0.26)	1.27 (0.89, 1.65)	2	260.8	267.5
B: plus local PCT using two PCA scores	128.4	0.77 (0.71, 0.83)	0.38	0.35	1.61 (0.26)	1.51 (0.26)	1.28 (0.89, 1.66)	4	264.8	278.2
D: plus local PCT using two individual measures (BF, BV)	128.3	0.76 (0.70, 0.82)	0.37	0.34	1.57 (0.25)	1.46 (0.26)	1.27 (0.89)	4	264.6	278.1
D2: plus local PCT using four individual measures (BF, BV, PS, and MTT)	128.1	0.77 (0.71, 0.82)	0.39	0.33	1.63 (0.26)	1.45 (0.26)	1.32 (0.92, 1.72)	6	268.2	288.4
E: plus central PCT using two PCA scores	127.7	0.78 (0.72, 0.84)	0.38	0.35	1.60 (0.25)	1.49 (0.25)	1.20 (0.84, 1.56)	4	263.3	276.8
F1: plus four main genes only	122.9	0.77 (0.71, 0.83)	0.47	0.41	1.94 (0.28)	1.72 (0.279)	1.25 (0.91, 1.60)	7	259.7	283.2
F2: plus IHC (CD105, HIF1, and MMR)	127.2	0.78 (0.72, 0.84)	0.41	0.37	1.70 (0.26)	1.55 (0.264)	1.31 (0.92, 1.70)	5	264.3	281.1
F3: plus four main genes and IHC (CD105, HIF1, and MMR)	121.9	0.78 (0.72, 0.84)	0.50	0.41	2.04 (0.30)	1.70 (0.29)	1.26 (0.92, 1.60)	10	263.8	297.3

*AIC* akaike information criterion, *BIC* Bayesian information criterion, *PCT* CT perfusion imaging, *PCA* principal components analysis, *IHC* immunohistochemistry, *BF* blood flow, *BV* blood volume, *PS* permeability surface, *MTT* mean transit time, *MMR* mismatch repair, *HIF* hypoxia-inducible factor, *SE* standard error

## Discussion

Prognostication in clinical practice is most commonly by AJCC staging [12], which combines tumour, nodal, and metastatic status. This is validated and widely accepted, but ignores additional potentially useful prognostic information [8]. Multivariable prognostic models in healthcare combine multiple factors to estimate the risk of future outcome(s), such as recurrence or death, and aim to inform clinical decisions by facilitating personalised management [14].

Models are typically developed using multivariable regression, which combines weighted predictors in an equation that estimates individual risk. Models previously proposed for colorectal cancer include Numeracy by Adjuvant! Online [15]. Novel markers promise to improve prognostication and to personalise the treatment of cancer patients, but a challenge for biomarker research, including imaging, immunohistochemical and genetic biomarkers, is limited power, over-optimistic prediction, and lack of generalisability of data from an investigation of single markers, small samples, and lack of prospective multicentre evaluation.

In this prospective multicentre trial, we verified that the sensitivity and specificity of TNM staging alone for the primary outcome (recurrence/death by 3 years), were limited at 0.56 and 0.58, respectively. In comparison to TNM staging, a clinicopathologic model including sex, age, tumour, and nodal stage, tumour location and size, vascular invasion and treatment improved specificity (0.74 vs 0.58) with equivalent sensitivity (0.57 vs 0.56) when used to identify high vs medium/low-risk participants. When used to identify high/medium vs low-risk patients, sensitivity was higher (0.89 vs 0.56), but with diminished specificity (0.40 vs 0.58). While this model was unable to simultaneously increase sensitivity and specificity substantially, it promises clinical utility by improving on prediction of recurrence compared to staging alone. Patients’ perspectives will influence which threshold to adopt; i.e. improved specificity to diminish overtreatment risk or improved sensitivity to diminish the chance of missing future recurrence.

In order to assess the prognostic utility of novel biomarkers, statisticians advocate building a “baseline” standard model from predictors already considered clinically useful [16], rather than selecting from the study dataset by univariable significance (which encourages overfitting) [17]. The benefit, if any, of promising biomarkers is then determined by whether their addition to the standard model improves prediction significantly, instead of continually re-fitting the entire model (which results in over-optimistic prediction) [18]. To avoid constraints imposed by retrospective datasets, we used a prospective design to eliminate recruitment biases and acquired sufficient events (namely patients developing distant metastasis or death). Evaluating multiple pre-specified predictors with adequate power necessitated a time-consuming multicentre design but ensured data represented were generalisable and represented up-to-date clinical practice.

However, we found that the addition of the prespecified promising CT perfusion imaging, genetic, and immunohistochemical markers to the clinicopathological model failed to improve prediction substantially over and above the baseline model. For example, when immunohistochemical/genetic variables were included with the clinicopathological variables, sensitivity and specificity at the high vs medium/low-risk threshold, were slightly higher (sensitivity, 0.68 vs 0.57; and specificity, 0.76 vs 0.74) but not to a clinically meaningful extent. For CT perfusion variables, sensitivity and specificity at the high vs medium/low-risk threshold were similar to the clinicopathological model (sensitivity, 0.58 vs 0.57; and specificity, 0.75 vs 0.74).

The belief that individual tumour biology influences prognosis, irrespective of stage, underpins recent extensive ‘omic’ research. For example, evidence suggests preoperative CT perfusion measures might predict subsequent recurrence by reflecting tumour angiogenesis and hypoxia [19–21]. RAS mutational testing may predict response to anti-epidermal growth factor receptor therapy and microsatellite instability or immunohistochemistry testing for MMR proteins to identify Lynch syndrome [22]. Accordingly, we hoped that these promising biomarkers of angiogenesis, hypoxia, and gene mutation would improve prediction.

That none of these pre-specified biomarkers improved prediction to a clinically relevant extent when added to the baseline clinicopathological model highlights the challenges for novel biomarker research. A recent article highlighted that ‘omic’ research often ignores clinical data and/or fails to develop models appropriately [23]. As proof, they developed a model for breast cancer survival that included stage, age, receptors, and grade. Adding gene expression failed to improve prediction and only became useful if clinical data were excluded altogether. Ultimately, the authors argued that omics, “may not be much more than surrogates for clinical data” [23]. Similarly, researchers found that predictors of cardiovascular disease contributed little over and above basic clinical measurements [24]. Expert opinion stipulated that our standard model includes extramural vascular invasion [25, 26], and we found extramural vascular invasion to be statistically significant in both univariable and multivariable analyses. Further research and models should consider including extramural vascular invasion, including CT imaging-assessed invasion in the neoadjuvant setting.

Our study has limitations. First, the number of participants developing distant metastasis or death was lower than expected from historical data (likely due to neoadjuvant therapy, improved surgery reducing resection margin positivity, and screening programmes that detect early-stage tumours), though target recruitment was achieved. Second, participants undergoing additional histopathological analysis were relatively small as our study was powered primarily for the CT imaging markers. Third, our findings should not be over-interpreted. While the baseline standard model was superior to standard current practice (AJCC staging), its clinical utility needs confirmation in daily practice. Finally, we made no comparison with commercial models (e.g. immunoscore [27]) that are used alongside TN staging.

In summary, we found that a prognostic model based on prospectively derived prespecified standard clinicopathological variables outperformed TN staging by either improving specificity or sensitivity, the latter at the cost of diminished specificity with promise for clinical practice. The addition of previously published promising imaging, immunohistochemical, and genetic biomarkers in a robust multicentre prospective trial did not substantially improve prediction performance, highlighting the potential of over-optimism of published prognostic markers.

## Supplementary information

Electronic Supplementary Material

## Abbreviations

AJCC: American Joint Committee on Cancer
CI: Confidence intervals
EMVI: Extramural vascular invasion
GLUT: Glucose transporter protein
HIF: Hypoxia inducible factor
TNM: Tumour-node-metastasis
VEGF: Vascular endothelial growth factor
