# Supplementary material for: Multivariable prognostic modelling to improve prediction of colorectal cancer recurrence: the PROSPeCT trial
Source: Eur Radiol. 2024 Jun 5;34(11):6992–7001. doi: 10.1007/s00330-024-10803-7 (PMC11519198; doi:10.1007/s00330-024-10803-7)
Supplement: Supplementary file 1 — Electronic Supplementary Material [file 330_2024_10803_MOESM1_ESM.pdf]

Multivariable prognostic modelling to improve prediction of colorectal cancer recurrence: The PROSPeCT trial

Electronic Supplementary Material (ESM)

Eur Radiol (2024) Goh V, Mallett S, Boulter V et al.

SUPPLEMENTARY MATERIAL.

Additional details regarding prognostic modelling.

The baseline model was a Wiebull parametric (STATA “stpm2”) with 1 degree-of-freedom (df), and proportional hazard assumptions checked. CT perfusion date was the reference date for calculation of date of recurrence. Date of recurrence was based on date of death or date of first scan showing recurrence, or no CT scan based on the date of the clinic visit where a decision of recurrence was made. Loss to follow up or censoring was based on the date of the last CT scan or last clinic visit.

The standard clinicopathologic model combined variables pre-specified based on the published literature and clinical opinion, consisting of tumour-node (TN) stage, age, sex, tumour location and size, extramural vascular invasion (EMVI), and planned treatment.

Model variables were selected based on the types of information that would be known at the time of model application. T stage, N stage and EMVI were from pathology reports, except where there was no baseline surgery or neoadjuvant treatment prior to surgery, which will modify pathological stage; in which case these variables were obtained from the imaging reports according to the following order: baseline CT staging, local perfusion CT, central perfusion CT. Where EMVI status was derived from central perfusion CT (either due to lack of alternative information or discordant results from pathology and local CT perfusion) interpretation was blinded to recurrence status.

Where possible, continuous variables were retained during modelling (age, tumour size) and modelled using linear relationships (after checking whether fit was improved using fractional polynomials) and centred around median values of 65 years and 40mm tumour size. Categorical variables used the following categories in modelling: tumour location as left or right colon – defined as right (reference category: corresponds to caecum, ascending colon, transverse colon without flexure or with hepatic flexure), and left (transverse colon if splenic flexure, descending colon, sigmoid colon) and rectosigmoid/rectum; T stage was included as four categories – T1, T2 (reference), T3 and T4; N stage was included as 3 categories –

N0 no lymph nodes (LN) positive, N1 one LN positive, N2 two or more LN positive; EMVI was categorised as positive or negative; treatment was grouped as 4 categories based on treatment plan at baseline imaging – surgery only, neoadjuvant therapy followed by surgery, surgery followed by adjuvant therapy, and no surgery; sex as male or female.

There was no variable selection in model development or validation (no univariable selection based on significance and no selection within model development). All model variables were pre-specified and retained in the final baseline model even if non-significant in univariable analysis. This was because significance is possible in different bootstrap versions of the model, and because these standard variables would be easily obtained in the course of normal clinical practice.

CT perfusion measured 4 parameters (blood flow per unit volume, blood volume, permeability surfaces area, mean transit time). Principal components analysis (PCA) was used to combine variables from CT perfusion for inclusion in modelling. Each CT perfusion parameters was standardized to a mean of 0 and a variance of 1, to avoid undue influence due to differences in measurement scales (STATA pca commands). We retained two principal component composite variables with eigenvalues of greater than one approximating to 70% of cumulative total variation.

Principal component scores were calculated (STATA predict pc1 pc2, score) and the contribution of CT perfusion to prediction of recurrence was based on looking for improved model prediction (sensitivity and specificity) and model fit (AIC, BIC) of nested models, when CT perfusion was added to an offset linear predictor from Model A (baseline clinical and imaging characteristics).

As a sensitivity analysis, CT perfusion variables were included as continuous linear variables without use of PCA. Genetic and immunohistochemistry variables were similarly investigated for their added predictive value compared to a Model A offset linear predictor, by including as predefined separate groups (two groups: genetic and immunohistochemistry) and as combining two groups.

324 of 326 participants had complete data for conventional variables and were used for Rule C and to develop Model A. While more participants had missing CT and

pathology variables, multiple imputation was not used because comparison was always comparing models within patients, so use of imputation would not increase statistical power [1].

Sample size justification

It was estimated that 20% of participants would have metastasis at presentation and 30% would develop metastasis within 36 months [2; 3]. It was estimated that 320 patients (including 10% follow-up loss), with 80 events provided 80% power to detect a 15% difference in correct risk classification between models, based on a reclassification index similar to Pencina [4]. Model variables were pre-specified to conserve statistical power in model development. Due to higher than anticipated withdrawals at staging, sample size increased to 445 following Independent Data Monitoring Committee advice. Recruitment ceased when the original event target was achieved.

Model A (clinicopathological variables) equation

Model A log cumulative hazard = -2.617 + 0.833*ln(time in years)

- 0.174&T1 + 0*T2 + 0.604*T3 + 1.739*T4 + 0*N0 + 0.604*N1 + 0.279*N2

+ 0*male -0.262*female + 0*NoEMVI + 0.657*EMVI + 0*left_colon + 0.421*right_colon

+ 0*surgery_only -0.393*NeoAdj_surgery - 0.110*Surgery_adjuvant + 1.582*No_surgery

+ 0*65years +0.045*increased_years_above_65yrs

+ 0*40mm_tumor_size -0.010*each_1mm_increased_size_over_40mm

Note: Model A used flexible parametric survival analysis based on stmp2 in STATA 14.1. The baseline hazard was selected based on comparing the smoothed baseline hazard from a Cox model to parametric baselines based on one to six degrees of freedom, which included zero to five spline knots in the baseline.

Modelling using one degree of freedom was chosen, which is a Wiebull model with no spline knots, based on the lowest AIC and BIC. This also was a more credible baseline reflecting the biological rate of recurrence, as opposed to peaks that artificially reflect timing of regular yearly CT imaging when the majority of recurrences were detected. The baseline model included a constant term and a shape parameter (rsc) which is multiplied by the natural log of time in years.

Models were fitted on the log cumulative hazard scale (ln(-ln S(t))) with proportional hazards. Nested models of Model A coefficients were fitted using a fixed offset of the Model A linear predictor, allowing the effect of additional variables to be assessed. Continuous variables were centred on values close to the median: age in years was centred for 65 years and tumor size was centred at 40mm.

References

1 Vergouwe Y, Royston P, Moons KG, Altman DG (2010) Development and validation of a prediction model with missing predictor data: a practical approach. J Clin Epidemiol 63:205-214

2 Sargent DJ, Patiyil S, Yothers G et al (2007) End points for colon cancer adjuvant trials: observations and recommendations based on individual patient data from 20,898 patients enrolled onto 18 randomized trials from the ACCENT Group. J Clin Oncol 25:4569-4574

3 Goh V, Halligan S, Wellsted DM, Bartram CI (2009) Can perfusion CT assessment of primary colorectal adenocarcinoma blood flow at staging predict for subsequent metastatic disease? A pilot study. Eur Radiol 19:79-89

4 Pencina MJ, D'Agostino RB, Sr., D'Agostino RB, Jr., Vasan RS (2008) Evaluating the added predictive ability of a new marker: from area under the ROC curve to reclassification and beyond. Stat Med 27:157-172; discussion 207-112

Supplementary Figure

Supplementary Figure 1. Kaplan-Meier curves for high vs. low-risk patients as defined by American Joint Committee on Cancer (AJCC) stage group (Rule C).

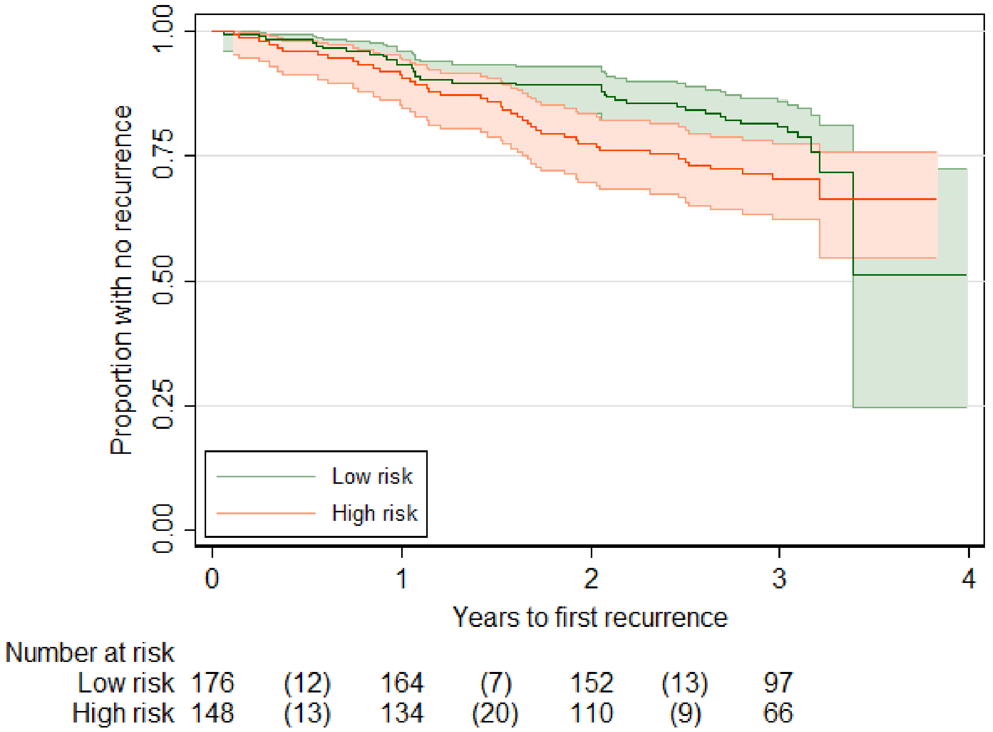

Supplementary Tables

Supplementary Table 1. Recommended perfusion CT acquisition parameters

Sequence	Topogram	Low dose planning sequence	Dynamic acquisition
**Siemens**	Spiral	Spiral	Dynserio 7.2
kV	120	100	100
mA<br>BMI <30	36	130<br>with tube current modulation	130<br>without tube current modulation
mA<br>BMI >30	36	150<br>with tube current modulation	150<br>without tube current modulation
Rotation time	-	0.5s	0.5s
Detector configuration	-	24X1.2mm	24X1.2mm
Slice collimation	0.6mm	5mm	7.2mm
Temporal interval /Length of scan	Topogram length 256mm-	Pitch 1.2<br>Direction craniocaudal	Cycle time<br>1.5/15seconds/120seconds
Reconstructed FOV		380mm	380 mm
Reconstruction kernel	-	B30f medium smooth	B30f medium smooth
Reconstruction slice thickness	-	5mm	7.2mm (<64MDCT)<br>5 mm (>64 MDCT)
**GE**	Spiral	Spiral	Axial mode
kV	120	100	100
mA<br>BMI <30	30	80<br>with tube current modulation	80<br>without tube current modulation
mA<br>BMI >30	30	100<br>with tube current modulation	100<br>without tube current modulation
Rotation time		0.5s	0.5s
Detector configuration			4*5mm<br>8*5mm
Slice collimation	0.6mm	5mm	5mm
Temporal interval /Length of scan	Topogram length 256mm-	Pitch 1.2<br>Direction craniocaudal	Cycle time<br>1.5/15 seconds/120seconds

Reconstruction kernel		B30 soft	B30 soft
Reconstruction slice thickness		2.5mm<br>5mm	2.5mm<br>5mm
**Toshiba**			
kV		100	100
mA		100	100
Rotation time		0.5s	0.5s
Detector configuration		320*0.5	320*0.5
Slice collimation		0.5mm	0.5mm
Temporal interval /Length of scan			Cycle time<br>1.5/15 seconds/120seconds
Reconstruction kernel		B30 soft	B30 soft
Reconstruction slice thickness		5mm	5mm

Supplementary Table 2. Local site perfusion CT measurements for participants with and without recurrence (available for 303/326 [93%] participants). There was no difference in measures between groups.

**Local review**	**With recurrence**		**Without recurrence**	
**Variable**	**Number of participants with data**	**Mean (SD)<br>Median (IQR)<br>Range**	**Number of participants with data**	**Mean (SD)<br>Median (IQR<br>Range**
Blood flow (mL/min/100mL or 100g)	78	72.9 (40.4)<br>62.5 (52.6, 85.1)<br>27.5, 350.8	225	69.2 (35.7)<br>63.1 (47.3, 81.9)<br>0, 248.0
Blood volume (mL/100mL or 100g)	76	13.1 (8.4)<br>11.3 (6.5, 16.3)<br>0.6, 46.7	220	12.9 (7.4)<br>12.5 (6.8,16.5)<br>0, 45.5
Permeability surface area product (mL/min/100mL or 100g)	70	18.1 (14.9)<br>13.8 (8.8, 19.9)<br>0.3, 66.8	199	16.5 (13.4)<br>12.8 (8.9, 18)<br>0, 72.1
Mean transit time (seconds)	68	13.2 (5.8)<br>11.6 (9, 17.7)<br>4.4, 29.7	186	13.8 (5.8)<br>13.3 (9.2, 18.1)<br>3.4, 33.6

Supplementary Table 3. Immunohistochemical scores for participants with and without recurrence.

Variable Score	With recurrence	Without recurrence	Total
**Hif-1α**	**N (%)**	**N (%)**	**N (%)**
0	18 (29)	58 (27)	76 (28)
1	5 (8)	24 (11)	29 (11)
2	11 (18)	35 (17)	46 (17)
3	1 (2)	4 (2)	5 (2)
4	11 (18)	40 (19)	51 (19)
6	15 (24)	46 (22)	61 (22)
Missing	1 (2)	5 (2)	6 (2)
**VEGF**	**N (%)**	**N (%)**	**N (%)**
0	55 (89)	199 (94)	254 (93)
2	2 (3)	4 (2)	6 (2)
3	2 (3)	3 (1)	5 (2)
4	2 (3)	2 (1)	4 (1)
Missing	1 (2)	4 (2)	5 (2)
**Glut1**	**N (%)**	**N (%)**	**N (%)**
0	2 (3)	10 (5)	12 (4)
1	0 (0)	1 (0)	1 (0)
2	0 (0)	5 (2)	5 (2)
3	2 (3)	8 (4)	10 (4)
4	4 (6)	13 (6)	17 (6)
5	5 (8)	18 (8)	23 (8)
6	18 (29)	56 (26)	74 (27)
7	10 (16)	46 (22)	56 (20)
8	20 (32)	51 (24)	71 (26)
Missing	1 (2)	4 (2)	5 (2)

Supplementary Table 4. Genetic mutation frequency for participants with and without recurrence.

Gene mutation	With recurrence	Without recurrence	Total
**MMR**	**N (%)**	**N (%)**	**N (%)**
Deficient	5 (8)	16 (8)	21 (8)
Proficient	56 (90)	192 (91)	248 (91)
Missing data	1 (2)	4 (2)	5 (2)
**KRAS**	**N (%)**	**N (%)**	**N (%)**
KRAS wild type	34 (55)	96 (45)	130 (47)
KRAS mutation	28 (45)	112 (53)	140 (51)
Missing data	0 (0)	4 (2)	4 (1)
**NRAS**	**N (%)**	**N (%)**	**N (%)**
NRAS wild type	55 (89)	188 (89)	243 (89)
NRAS mutation	7 (11)	20 (9)	27 (10)
Missing data	0 (0)	4 (2)	4 (1)
**HRAS**	**N (%)**	**N (%)**	**N (%)**
HRAS wild type	52 (84)	184 (87)	236 (86)
HRAS mutation	10 (16)	24 (11)	34 (12)
Missing data	0 (0)	4 (2)	4 (1)
**BRAF V600E**	**N (%)**	**N (%)**	**N (%)**
BRAF V600E wild type	59 (95)	192 (91)	251 (92)
BRAF V600E mutation	3 (5)	16 (8)	19 (7)
Missing data	0 (0)	4 (2)	4 (1)
**BRAF other**	**N (%)**	**N (%)**	**N (%)**
BRAF other wild type	53 (85)	172 (81)	225 (82)
BRAF other mutation	9 (15)	36 (17)	45 (16)
Missing data	0 (0)	4 (2)	4 (1)

Supplementary Table 5. Univariable hazard ratios for standard clinicopathological variables included in Model A.

Variable	N	HR (95% CI)	p-value*
T stage			
T1	324	0.61 (0.08 to 4.6)	<0.001
T2 (reference)	324	1 (1 to 1)	
T3	324	2.01 (1.06 to 3.79)	
T4	324	5.48 (2.72 to 11)	
N stage			
N1 (1 LN positive)	324	1.22 (0.74 to 2.03)	0.11
N2 (2 or more positive)	324	1.82 (1.04 to 3.19)	
EMVI	324	2.37 (1.53 to 3.67)	<0.001
Treatment group (reference group is Surgery only)			<0.001
Neoadjuvant therapy +Surgery	324	1.03 (0.56 to 1.89)	
Surgery + adjuvant therapy	324	1.11 (0.65 to 1.91)	
No surgery	324	6.12 (3.14 to 11)	
Rectal (ref Left Colon)	324	0.95 (0.61 to 1.47)	0.81
Right colon location	324	1.81 (1.1 to 2.98)	0.02
Tumour size (mm)	324	1.00 (0.99 to 1.01)	0.96
Age (years)	324	1.06 (1.03 to 1.08)	<0.001
Sex (male reference)	324	1.01 (0.63 to 1.62)	0.96

* Overall Wald test for categorical data of more than two categories
* Left colon location is reference. Mid location is grouped with left

Supplementary Table 6. Multivariable hazard ratios for standard clinicopathological variables included in Model A.

Variable	N	HR (95% CI)	p-value*
T stage			
T1	324	0.84 (0.11 to 6.52)	<0.001
T2 (reference)	324	1 (1 to 1)	
T3	324	1.83 (0.90 to 3.70)	
T4	324	5.69 (2.52 to 12)	
N stage			
N1 (1 LN positive)	324	1.10 (0.60 to 2.01)	0.69
N2 (2 or more positive)	324	1.32 (0.70 to 2.51)	
EMVI	324	1.93 (1.17 to 3.19)	0.01
Treatment group (reference group is Surgery only)			<0.001
Neoadjuvant therapy + surgery	324	0.67 (0.35 to 1.30)	
Surgery + adjuvant therapy	324	0.90 (0.42 to 1.92)	
No surgery	324	4.87 (2.37 to 10)	
Right colon location (left is reference)	324	1.52 (0.88 to 2.65)	0.14
Tumour size (mm)	324	0.99 (0.98 to 1.00)	0.19
Age (years)	324	1.05 (1.02 to 1.07)	<0.001
Sex (male reference)	324	0.77 (0.47 to 1.26)	0.30
Rcs1	324	2.30 (1.95 to 2.71)	
Constant	324	0.07 (0.04 to 0.14)	

* overall Wald test for categorical data of more than two categories
* Age is centred at 65 years and tumour size is centred at 40mm. Constant term when Model A is not centred is 0.006

Supplementary Table 7. Univariable hazard ratios for immunohistochemical and somatic mutation variables.

Variables	N	HR (95% CI)	p-value
CD105	253	1.00 (0.99 to 1.00)	0.83
Hif1a**	253	1.02 (0.61 to 1.71)	0.94
MMR	253	0.85 (0.34 to 2.11)	0.72
BRAF not 600	253	0.67 (0.30 to 1.48)	0.32
BRAF 600	253	0.69 (0.22 to 2.20)	0.53
KRAS	253	0.71 (0.42 to 1.19)	0.19
HRAS	253	1.34 (0.63 to 2.82)	0.44
NRAS	253	1.33 (0.60 to 2.92)	0.48

**binary form Hif1a_2w 0=0-2, 1=3-6
* MMR proficient compared to a reference standard of deficient. Deficient refers to tumour with mutation.

Supplementary Table 8. Multivariable hazard ratios for immunohistochemical and somatic mutation variables.

	N	HR (95% CI)	p-value
ModelA_323	212	2.718 constrained	
CD105	212	1.00 (0.99 to 1.01)	0.56
MMR*	212	0.64 (0.24 to 1.68)	0.36
Hif1a**	212	1.33 (0.74 to 2.34)	0.34
BRAF not 600	212	0.57 (0.23 to 1.40)	0.22
BRAF 600	212	0.34 (0.08 to 1.50)	0.16
KRAS	212	0.57 (0.31 to 1.06)	0.08
HRAS	212	1.99 (0.88 to 4.55)	0.10
NRAS	212	2.32 (0.93 to 5.78)	0.07
baseline survival (rcs1)	212	2.19 (1.79 to 2.69)	<0.001
Constant	212	0.01 (0.004 to 0.05)	<0.001
